# Anastomotic Leak Does Not Impact on Long-Term Outcomes in Esophageal Cancer Patients

**DOI:** 10.1245/s10434-020-08199-x

**Published:** 2020-01-23

**Authors:** S. K. Kamarajah, M. Navidi, S. Wahed, A. Immanuel, N. Hayes, S. M. Griffin, A. W. Phillips

**Affiliations:** 1 Northern Oesophagogastric Unit, Royal Victoria Infirmary, Newcastle University Trust Hospitals, Newcastle upon Tyne, UK; 2 Institute of Cellular Medicine, Newcastle University, Newcastle upon Tyne, UK; 3 School of Medical Education, Newcastle University, Newcastle upon Tyne, UK

## Abstract

**Background:**

Esophagectomy is a technically demanding procedure associated with high levels of morbidity. Anastomotic leak (AL) is a common complication with potentially major ramifications for patients. It has also been associated with poorer long-term overall survival (OS) and disease recurrence.

**Objective:**

The aim of this study was to determine whether AL contributes to poor OS and recurrence-free survival (RFS) for patients with esophageal cancer.

**Methods:**

Consecutive patients undergoing a two-stage, two-field transthoracic esophagectomy from a single high-volume unit between 1997 and 2016 were evaluated. Clinicopathologic characteristics, along with oncological and postoperative outcomes, were stratified by no AL versus non-severe leak (NSL) versus severe esophageal AL (SEAL). SEAL was defined as ALs associated with Clavien–Dindo grade III/IV complications.

**Results:**

This study included 1063 patients, of whom 8% (87/1063) developed AL; 45% of those who developed AL were SEALs (39/87). SEAL was associated with a prolonged critical care stay (median 8 vs. 3 vs. 2 days; *p* < 0.001) and prolonged hospital stay (median 43 vs. 27 vs. 15 days; *p* < 0.001) compared with NSL or no AL. There were no significant differences in number of lymph nodes harvested and rates of R1 resection between groups. OS and RFS were not affected by either NSL or SEAL, and Cox multivariate regression showed NSL and SEAL were not independently associated with OS and RFS. Sensitivity analysis in patients receiving neoadjuvant therapy followed by esophagectomy demonstrated similar findings.

**Conclusion:**

These results demonstrate that AL leads to prolonged critical care and in-hospital length of stay; however, contrary to previous reports, our results do not compromise long-term outcomes and are unlikely to have a detrimental oncological impact.

Esophagectomy remains a key component of treatment for patients with potentially curable esophageal cancer. While mortality levels from the procedure have fallen dramatically over the last 30 years, esophagectomy is still associated with high levels of morbidity.^1^^–^3 Anastomotic leak (AL) is a commonly seen complication that has historically been associated with high mortality rates.^4^ The Esophagectomy Complications Consensus Group (ECCG) defined AL as a full-thickness defect involving the esophagus, anastomosis, staple line or conduit, irrespective of the presentation or method of identification.^5^ The classification further divided leaks into the management strategy employed: type I, those that require no change in treatment; type II, leaks that require intervention, but not surgery; and type III, leaks that require surgical intervention.

The incidence of AL has been reported at between 3 and 30%.^6^^,^^7^ This can result in a prolonged hospital stay, a need for reoperation, anastomotic stricturing that requires repeated dilations, and potentially poorer long-term survival.^8^^,^^9^ A French multicenter study, which defined severe esophageal leaks (SEALs) as those that equated to a grade III/IV Clavien–Dindo complication, demonstrated poor long-term prognosis in patients who developed SEAL following esophagectomy.^9^ This study was limited by variations in oncological and surgical pathways and included a mixture of high- and low-volume units. Furthermore, this study did not report on recurrence-free survival (RFS).

Despite the above study, the impact of AL is unclear, with conflicting evidence, and the majority of the published literature are limited by small series.^4^^,^^9^^–^12 The aim of this study was to evaluate outcomes from a single high-volume center and to determine whether AL impacts on oncological and postoperative outcomes as well as long-term overall survival (OS) and RFS.

## Methods

### Patient Population

Consecutive patients from the Northern Oesophagogastric Unit, Newcastle upon Tyne, treated for adenocarcinoma or squamous cell carcinoma (SCC) of the esophagus or gastroesophageal junction between January 1997 and December 2016 were included. All patients were discussed at a multidisciplinary meeting and subsequently received neoadjuvant chemo(radio)therapy followed by transthoracic esophagectomy (Ivor Lewis). Patients were identified from a contemporaneously maintained database.

### Pretreatment Staging

All patients were staged according to standardized protocols, which included endoscopy with biopsy, endoscopic ultrasonography, external ultrasonography of the neck (if required), and a thoracoabdominal computed tomography (CT) scan. A positron emission tomography (CT) scan is used in patients being considered for radical (curative) treatment. In patients with histology proven, locally advanced resectable malignancy without metastases (cT1N + or cT3N0-3) or tumors of questionable resectability (cT4), neoadjuvant chemo(radio)therapy followed by surgery is the main treatment option. Patients with a histology other than adenocarcinoma or SCC and metastatic disease at the time of operation were excluded.

### Treatment

Multiple neoadjuvant regimens were employed in the present study, determined by the standard of care and recruiting clinical trials at the time of treatment (Table 1), with patients treated earlier in the time period having unimodality surgery. The majority of patients treated received neoadjuvant chemotherapy. Transthoracic esophagectomy with two-field lymph node dissection was performed within 4–8 weeks after completion of the neoadjuvant therapy using a conventional or minimally invasive approach as previously described.^13^

**Table 1:** Clinicopathologic characteristics of patients undergoing esophagectomy for esophageal cancer

	Overall [*n* =1063]	No AL [*n* =976]	AL [*n* =48]	SEAL [*n* =39]	*p* value
Age at presentation, years	65 (58–71)	65 (58–71)	64 (60–71)	66 (60–72)	0.700
Sex, male	811 (76)	739 (76)	41 (85)	31 (79)	0.271
Histology, SCC	207 (19)	190 (19)	12 (25)	5 (13)	0.361
BMI, kg/m^2^	26 (24–29)	26 (24–29)	27 (23–30)	26 (24–29)	0.777
Smoking status					0.302
Current	260 (24)	233 (24)	17 (35)	10 (26)	
Ex-smoker	493 (46)	449 (46)	23 (48)	21 (54)	
Never	302 (28)	286 (29)	8 (17)	8 (21)	
Unknown	8 (1)	8 (1)	0 (0)	0 (0)	
Alcohol status					0.009
Current	770 (72)	703 (72)	34 (71)	33 (85)	
Ex-drinker	75 (7)	63 (6)	9 (19)	3 (8)	
Never	199 (19)	191 (20)	5 (10)	3 (8)	
Unknown	19 (2)	19 (2)	0 (0)	0 (0)	
ASA grade					0.309
1	158 (15)	145 (15)	9 (19)	4 (10)	
2	539 (51)	496 (51)	19 (40)	24 (62)	
3	270 (25)	247 (25)	16 (33)	7 (18)	
4	7 (1)	5 (1)	1 (2)	1 (3)	
Unknown	89 (8)	83 (9)	3 (6)	3 (8)	
Overall treatment, surgery only	500 (47)	458 (47)	17 (35)	25 (64)	0.028
Neoadjuvant chemotherapy regimen					0.035
CF	208 (20)	184 (19)	17 (35)	7 (18)	
CROSS	20 (2)	18 (2)	2 (4)	0 (0)	
ECF/ECX	285 (27)	267 (27)	11 (23)	7 (18)	
None	500 (47)	458 (47)	17 (35)	25 (64)	
Unknown	50 (5)	49 (5)	1 (2)	0 (0)	
Overall AJCC 8th edition pathological stage					0.311
0	49 (5)	45 (5)	3 (6)	1 (3)	
I	243 (23)	223 (23)	8 (17)	12 (31)	
II	234 (22)	209 (21)	12 (25)	13 (33)	
III	450 (42)	415 (43)	23 (48)	12 (31)	
IV	87 (8)	84 (9)	2 (4)	1 (3)	
Tumor grade					0.222
Well	94 (9)	87 (9)	2 (4)	5 (13)	
Moderate	512 (48)	475 (49)	18 (38)	19 (49)	
Poor	393 (37)	353 (36)	26 (54)	14 (36)	
Unknown	64 (6)	61 (6)	2 (4)	1 (3)	
Lymph nodes harvested	30 (23–39)	30 (23–39)	30 (22–39)	26 (20–37)	0.335
Margin status, R1	26 (2)	23 (2)	1 (2)	2 (5)	0.539
Lymphatic involvement	490 (46)	452 (46)	24 (50)	14 (36)	0.378
Venous involvement	373 (35)	347 (36)	19 (40)	7 (18)	0.062
Perineural involvement	476 (45)	442 (45)	23 (48)	11 (28)	0.099
Tumor regression grade					0.502
1	38 (4)	35 (4)	2 (4)	1 (3)	
2	30 (3)	29 (3)	1 (2)	0 (0)	
3	79 (7)	72 (7)	5 (10)	2 (5)	
4	163 (15)	151 (15)	10 (21)	2 (5)	
5	44 (4)	40 (4)	3 (6)	1 (3)	
Unknown	709 (67)	649 (66)	27 (56)	33 (85)	
Extracapsular spread	179 (17)	162 (17)	10 (21)	7 (18)	0.733
Critical care stay, days	2 (1–5)	2 (1–5)	3 (2–8)	8 (3–18)	< 0.001
Total hospital stay, days	15 (12–22)	15 (12–21)	27 (15–40)	43 (32–64)	< 0.001
Overall complications	709 (67)	622 (64)	48 (100)	39 (100)	< 0.001
Surgical site infection	108 (10)	100 (10)	4 (8)	4 (10)	0.912
Pulmonary complications	123 (12)	109 (11)	9 (19)	5 (13)	0.268
Cardiac complications	73 (7)	65 (7)	5 (10)	3 (8)	0.591
Anastomotic leaks	87 (8)	0 (0)	48 (100)	39 (100)	
In-hospital mortality	38 (4)	34 (3)	1 (2)	3 (8)	0.324
30-day mortality	28 (3)	26 (3)	0 (0)	2 (5)	0.325

*AL* anastomotic leaks, *ASA* American Society of Anesthesiologists, *SEAL* severe esophageal AL, *SCC* squamous cell carcinoma, *BMI* body mass index, *AJCC* American Joint Committee on Cancer, *CF* Cisplatin and 5-Fluorouracil, *CROSS* Carboplatin, Paclitaxel and Radiotherapy, *ECF* Epirubicin, Cisplatin, 5-Fluorouracil, *ECX* Epirubicin, Cisplatin, Capecitabine

### Pathology and Staging

Histopathological reporting was carried out by specialist gastrointestinal pathologists using a standardized proforma. This was in line with guidelines produced by the Royal College of Pathologists, which included tumor type and differentiation, depth of tumor infiltration, and tumor regression.^14^ The total number of nodes from each location, as well as nodal metastases, were recorded, along with the presence of extracapsular, lymphatic, and venous and perineural invasion. Lymph nodes were dissected from the specimen by the operating surgeon and analyzed separately by the pathologist.^15^ The pathological stage was determined using the American Joint Committee on Cancer (AJCC) 8th edition TNM staging system.^16^

### Definition of Anastomotic Leak

AL was defined as a full-thickness gastrointestinal defect involving the esophagus, anastomosis, staple line, or conduit irrespective of presentation or method of identification according to the ECCG criteria. Type I AL was defined as a local defect requiring no change in therapy, or treated medically or with dietary modification; type II AL was defined as a localized defect requiring interventional but not surgical therapy, for example interventional radiology drain, stent or bedside opening, and packing of incision; and type III AL was defined as a localized defect requiring surgical therapy. In this study, patients with AL associated with grade III–V complications, as defined by the Clavien–Dindo 17 grading system, were defined as severe esophageal AL (SEAL), and those with less severe complications (Clavien–Dindo grade I/II were classified as non-severe leaks [NSLs]). Postoperative contrast swallows were not routinely used to determine if a leak was present and investigations were performed if there was clinical concern.

### Follow-Up and Definition of Recurrence

Patients were followed up until death or for 10 years. Patients were seen at 3- to 6-monthly intervals in the first 2 years, 6-monthly for 2 years, and then annually. Recurrence of disease was based on clinical grounds and was confirmed endoscopically or radiologically. The minimum follow-up was 30 months.

### Statistical Analysis

Categorical variables were compared using the Chi-square test; non-normally distributed data were analyzed using the Mann–Whitney *U* test; survival was estimated using Kaplan–Meier survival curves and compared using the log-rank test; and multivariable analyses used Cox proportional hazards models. A subset analysis in patients receiving neoadjuvant therapy prior to esophagectomy were analyzed. A *p* value < 0.05 was considered to be statistically significant. Data analysis was performed using R Foundation Statistical software (R 3.2.2) with TableOne, ggplot2, Hmisc, Matchit and survival packages (R Foundation for Statistical Computing, Vienna, Austria) as previously described.^18^^,^^19^

## Results

### Overall Cohort

#### Baseline Demographics

This study included 1063 patients undergoing esophagectomy for esophageal cancer, of whom 563 (53%) received neoadjuvant therapy. Clinicopathological variables are presented in Table 1. The median age of the entire cohort was 65 years (interquartile range 58–71 years), 76% were male, and 19% had SCC. Of the 1063 patients, 8% developed ALs (87/1063), of whom 45% (39/87) developed SEAL. Two patients who developed AL (2%) died.

Across the groups, there were no significant differences in age, sex, and rates of SCC; however, those developing SEAL were more likely to receive unimodality surgery (64%) compared with those with no ALs (47%) or NSL only (35%) [*p* = 0.028]. There were no significant differences in the rates of advanced tumor stage, number of lymph nodes examined, and R1 margins.

#### Postoperative Outcomes

Patients developing SEAL had a significantly longer length of stay in critical care (median 8 vs. 3 vs. 2 days; *p* < 0.001) and overall hospital stay (median 43 vs. 27 vs. 15 days; *p* < 0.001) compared with NSL or no AL. However, there were no significant differences in the rates of surgical site infections, cardiac complications, pulmonary complications, and in-hospital mortality across these groups.

#### Overall and Recurrence-Free Survival

**Fig. 1:**
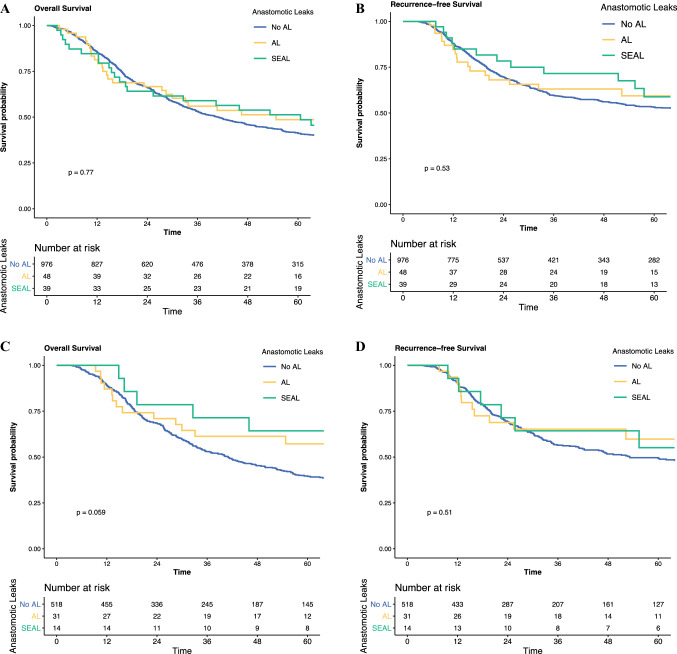
Impact of SEAL on OS and RFS in all patients and neoadjuvant therapy and surgery only. **a** OS in all patients; **b** RFS in all patients; **c** OS in NAT and surgery and **d** RFS in NAT and surgery. *AL* anastomotic leak, *NR* not reached, *NSL* non-severe leak, *SEAL* severe esophageal AL, *OS* overall survival, *RFS* recurrence-free survival

There was no significant difference in OS between the groups, with patients experiencing SEAL having a median survival of 61 months, compared with 55 months for patients with NSL and 41 months for patients with no AL only (*p* = 0.8) (Fig. 1a). On Cox multivariate regression, both NSL only (hazard ratio [HR] 0.91; *p* = 0.6) and SEAL (HR 1.24; *p* = 0.3) were not independently associated with OS (Table 2). Patients developing SEAL had similar RFS as patients with NSL or no AL (Fig. 1b). On Cox multivariate regression, NSL (HR 0.86; *p* = 0.5) or SEAL (HR 1.19; *p* = 0.6) was not independently associated with OS (Table 2).

**Table 2:** Cox multivariate regression on the impact of SEAL on overall survival and recurrence-free survival

	Overall survival		Recurrence-free survival	
	HR (95% CI)	*p* value	HR (95% CI)	*p* value
Age at presentation	1.02 (1.01–1.03)	**<0.001**	1.00 (0.99–1.01)	0.991
Gender, male	1.34 (1.10–1.64)	**0.003**	1.39 (1.07–1.80)	**0.015**
ASA grade				
1	Ref		Ref	
2	1.17 (0.93–1.48)	0.189	0.89 (0.67–1.19)	0.442
3	1.30 (1.01–1.68)	**0.041**	0.99 (0.72–1.35)	0.93
4	1.96 (0.83–4.60)	0.124	0.37 (0.05–2.77)	0.335
Unknown	1.11 (0.81–1.54)	0.515	1.08 (0.72–1.62)	0.722
Histology, SCC	1.30 (1.04–1.61)	**0.019**	1.10 (0.82–1.48)	0.522
Operation year	0.96 (0.94–0.98)	**0.001**	0.97 (0.94–1.00)	**0.038**
Overall treatment, surgery only	1.14 (0.93–1.41)	0.213	1.12 (0.85–1.47)	0.419
Tumor grade				
Well	Ref		Ref	
Moderate	1.28 (0.92–1.78)	0.141	2.22 (1.21–4.08)	**0.01**
Poor	1.55 (1.09–2.19)	**0.014**	2.62 (1.41–4.87)	**0.002**
Unknown	1.73 (1.01–2.97)	**0.046**	2.70 (1.06–6.90)	**0.038**
Margin status, R1	2.05 (1.35–3.11)	**0.001**	1.72 (0.97–3.04)	0.065
Lymphatic involvement, yes	1.40 (1.15–1.71)	**0.001**	1.58 (1.23–2.04)	< 0.001
Venous involvement, yes	1.00 (0.83–1.22)	0.971	1.07 (0.84–1.36)	0.59
Perineural involvement, yes	1.42 (1.17–1.72)	**<0.001**	1.57 (1.23–2.00)	**<0.001**
Tumor regression grade				
1	Ref		Ref	
2	1.14 (0.48–2.71)	0.763	0.96 (0.32–2.85)	0.941
3	0.94 (0.45–1.95)	0.871	0.74 (0.30–1.83)	0.52
4	1.28 (0.66–2.49)	0.469	1.02 (0.44–2.35)	0.963
5	1.35 (0.65–2.79)	0.42	0.99 (0.40–2.47)	0.984
Unknown	1.25 (0.66–2.36)	0.498	0.82 (0.36–1.83)	0.625
Extracapsular spread, yes	1.71 (1.34–2.18)	**<0.001**	1.42 (1.06–1.91)	0.02
Overall AJCC 8th edition pathological stage				
0	Ref		Ref	
I	1.09 (0.59–2.00)	0.788	1.94 (0.58–6.41)	0.279
II	1.09 (0.59–2.00)	0.781	2.28 (0.70–7.45)	0.172
III	2.40 (1.31–4.40)	**0.005**	5.93 (1.82–19.28)	**0.003**
IV	3.57 (1.85–6.88)	**<0.001**	7.77 (2.30–26.25)	**0.001**
Anastomotic leaks				
No anastomotic leaks	Ref		Ref	
NSL	0.91 (0.62–1.35)	0.646	0.86 (0.52–1.40)	0.539
SEAL	1.24 (0.82–1.88)	0.302	1.19 (0.66–2.16)	0.561

*ASA* American Society of Anesthesiologists, *SEAL* severe esophageal anastomotic leak, *SCC* squamous cell carcinoma, *AJCC* American Joint Committee on Cancer, *HR* hazard ratio, *CI* confidence interval, *Ref* reference, *NSL* non-severe leak

Bold values indicate statistical significance (*P* <0.05)

### Neoadjuvant and Surgery

#### Baseline Demographics

In this subgroup analysis, 563 patients were included. Clinicopathological variables are presented in Table 3. Of these patients, 8% developed ALs (45/563), of whom 31% (14/45) developed SEAL. Across the groups, there were no significant differences in age, sex, and rates of SCC, and no significant differences in rates of advanced tumor stage, number of lymph nodes examined, and R1 margins.

**Table 3:** Clinicopathologic characteristics of patients undergoing esophagectomy following neoadjuvant therapy for esophageal cancer

	No AL [*n* =518]	AL [*n* =31]	SEAL [*n* =14]	*p* value
Age at presentation, years	64 (57–69)	63 (60–70)	66 (60–73)	0.581
Sex, male	407 (79)	26 (84)	13 (93)	0.346
Histology, SCC	101 (19)	10 (32)	1 (7)	0.108
BMI, kg/m^2^	26 (24–30)	28 (25–30)	26 (24–29)	0.702
Smoking status				0.169
Current	121 (23)	14 (45)	4 (29)	
Ex-smoker	246 (47)	11 (35)	8 (57)	
Never	146 (28)	6 (19)	2 (14)	
Unknown	5 (1)	0 (0)	0 (0)	
Alcohol status				0.274
Current	391 (75)	24 (77)	12 (86)	
Ex-drinker	31 (6)	4 (13)	2 (14)	
Never	92 (18)	3 (10)	0 (0)	
Unknown	4 (1)	0 (0)	0 (0)	
ASA grade				0.644
1	74 (14)	6 (19)	1 (7)	
2	292 (56)	12 (39)	9 (64)	
3	124 (24)	12 (39)	3 (21)	
4	2 (0)	0 (0)	0 (0)	
Unknown	26 (5)	1 (3)	1 (7)	
Neoadjuvant chemotherapy regimen				0.179
CF	184 (36)	17 (55)	7 (50)	
CROSS	18 (3)	2 (6)	0 (0)	
ECF/ECX	267 (52)	11 (35)	7 (50)	
Unknown	49 (9)	1 (3)	0 (0)	
Overall AJCC 8th edition pathological stage				0.683
0	30 (6)	2 (6)	1 (7)	
I	64 (12)	4 (13)	1 (7)	
II	135 (26)	8 (26)	7 (50)	
III	233 (45)	15 (48)	5 (36)	
IV	56 (11)	2 (6)	0 (0)	
Tumor grade				0.768
Well	17 (3)	1 (3)	1 (7)	
Moderate	251 (48)	12 (39)	6 (43)	
Poor	212 (41)	17 (55)	6 (43)	
Unknown	38 (7)	1 (3)	1 (7)	
Lymph nodes harvested	33 (26–42)	34 (26–40)	36 (30–39)	0.974
Margin status, R1	6 (1)	0 (0)	0 (0)	0.768
Lymphatic involvement	259 (50)	15 (48)	6 (43)	0.86
Venous involvement	190 (37)	11 (35)	2 (14)	0.227
Perineural involvement	245 (47)	14 (45)	5 (36)	0.679
Tumor regression grade				0.822
1	21 (4)	2 (6)	0 (0)	
2	28 (5)	1 (3)	0 (0)	
3	72 (14)	5 (16)	2 (14)	
4	148 (29)	10 (32)	2 (14)	
5	38 (7)	3 (10)	1 (7)	
Unknown	211 (41)	10 (32)	9 (64)	
Extracapsular spread	127 (25)	9 (29)	4 (29)	0.809
Critical care stay, days	2 (1–4)	4 (2–8)	8 (2–16)	0.001
Total hospital stay, days	14 (11–19)	27 (16–40)	37 (31–60)	< 0.001
Overall complications	316 (61)	31 (100)	14 (100)	< 0.001
Surgical site infection	45 (9)	2 (6)	3 (21)	0.226
Pulmonary complications	69 (13)	6 (19)	2 (14)	0.636
Cardiac complications	48 (9)	5 (16)	1 (7)	0.43
Anastomotic leaks	0 (0)	31 (100)	14 (100)	< 0.001
In-hospital mortality	15 (3)	0 (0)	0 (0)	0.512
30-day mortality	10 (2)	0 (0)	0 (0)	0.643

*AL* anastomotic leak, *ASA* American Society of Anesthesiologists, *HR* hazard ratio, *SEAL* severe esophageal AL, *SCC* squamous cell carcinoma, *BMI* body mass index, *AJCC* American Joint Committee on Cancer, *CF* Cisplatin and 5-Fluorouracil, *CROSS* Carboplatin, Paclitaxel and Radiotherapy, *ECF* Epirubicin, Cisplatin, 5-Fluorouracil, *ECX* Epirubicin, Cisplatin, Capecitabine

#### Postoperative Outcomes

Patients developing SEAL had significantly longer length of stay in critical care (median 8 vs. 4 vs. 2 days; *p* < 0.001) and overall hospital stay (median 37 vs. 27 vs. 14 days; *p* < 0.001) compared with NSL or no AL. However, there were no significant differences in the rates of surgical site infections, cardiac complications, pulmonary complications, and in-hospital mortality across these groups.

#### Overall and Recurrence-Free Survival

There was no significant difference in survival between cohorts (SEAL: median, not reported [NR] vs. NSL: 77 vs. no leak: 41 months; *p* = 0.058). On Cox multivariate regression, both NSL (HR 0.65; *p* = 0.1) and SEAL (HR 0.48; *p* = 0.09) were not independently associated with OS (Table 4). Patients developing SEAL had similar RFS as patients with NSL or no AL (Fig. 1d). On Cox multivariate regression, both NSL (HR 0.70; *p* = 0.3) or SEAL (HR 1.04; *p* = 0.9) were not independently associated with OS (Table 4).

**Table 4:** Cox multivariate regression on the impact of SEAL on overall survival and recurrence-free survival

	Overall survival		Recurrence-free survival	
	HR (95% CI)	*p* value	HR (95% CI)	*p* value
Age at presentation	1.01 (1.00–1.02)	0.118	1.00 (0.98–1.01)	0.753
Gender, male	1.41 (1.04–1.92)	**0.027**	1.21 (0.84–1.73)	0.306
ASA grade				
1	Ref		Ref	
2	0.95 (0.69–1.32)	0.77	0.90 (0.61–1.32)	0.581
3	1.00 (0.69–1.44)	0.987	0.95 (0.61–1.47)	0.821
4	0.95 (0.21–4.37)	0.952	0.53 (0.07–4.35)	0.557
Unknown	0.80 (0.45–1.42)	0.448	0.82 (0.41–1.64)	0.578
Histology, SCC	1.26 (0.91–1.75)	0.162	1.00 (0.66–1.51)	0.987
Operation year	0.97 (0.92–1.02)	0.19	0.96 (0.91–1.02)	0.206
Tumor grade				
Well	Ref		Ref	
Moderate	1.35 (0.68–2.68)	0.392	1.19 (0.54–2.59)	0.668
Poor	1.68 (0.84–3.36)	0.145	1.35 (0.61–2.98)	0.459
Unknown	1.09 (0.39–3.09)	0.868	1.10 (0.30–4.05)	0.887
Margin status, R1	1.33 (0.57–3.15)	0.511	1.73 (0.61–4.89)	0.303
Lymphatic involvement, yes	1.36 (1.02–1.80)	**0.034**	1.24 (0.88–1.73)	0.212
Venous involvement, yes	1.00 (0.76–1.31)	0.999	1.06 (0.77–1.47)	0.708
Perineural involvement, yes	1.51 (1.15–1.97)	**0.003**	1.81 (1.30–2.51)	**<0.001**
Tumor regression grade				
1	Ref		Ref	
2	0.94 (0.26–3.45)	0.93	0.23 (0.05–1.13)	0.07
3	0.84 (0.26–2.76)	0.779	0.30 (0.08–1.21)	0.09
4	1.21 (0.38–3.78)	0.748	0.44 (0.12–1.69)	0.235
5	1.33 (0.41–4.35)	0.637	0.45 (0.11–1.79)	0.255
Unknown	1.11 (0.35–3.53)	0.863	0.32 (0.08–1.23)	0.097
Extracapsular spread, yes	1.70 (1.26–2.28)	**<0.001**	1.40 (0.99–1.99)	0.057
Overall AJCC 8th edition pathological stage				
0	Ref		Ref	
I	0.74 (0.27–2.08)	0.572	3.98 (0.76–20.94)	0.103
II	0.55 (0.21–1.46)	0.23	2.51 (0.51–12.38)	0.259
III	1.43 (0.53–3.85)	0.474	7.01 (1.37–35.80)	**0.019**
IV	1.74 (0.61–4.98)	0.301	7.98 (1.49–42.91)	**0.015**
Anastomotic leaks				
No anastomotic leaks	Ref		Ref	
NSL	0.65 (0.39–1.09)	0.105	0.70 (0.38–1.30)	0.259
SEAL	0.48 (0.21–1.12)	0.09	1.04 (0.45–2.43)	0.919

*ASA* American Society of Anesthesiologists, *HR* hazard ratio, *CI* confidence interval, *NSL* non-severe leak, *Ref* reference, *SEAL* severe esophageal anastomotic leak, *SCC* squamous cell carcinoma, *AJCC* American Joint Committee on Cancer

Bold values indicate statistical significance (*P* <0.05)

## Discussion

The results of this study indicate that patients with AL, as well as those with severe ALs, do not have a poorer long-term survival than those patients who do not have an AL. In the short-term, NSL and SEAL were associated with a significantly longer stay in critical care and also longer time in hospital postoperatively. In-hospital mortality was not significantly different between those who had an AL and those who did not. In addition, there were comparable oncological outcomes between the groups in terms of R1 margin rates and number of lymph nodes harvested, which may reflect the similar survival between the groups, even after multivariable Cox regression analyses.

These findings are contrary to those of a previously published multicenter study that demonstrated that SEAL is associated with reduced long-term OS and worse recurrence rates.^9^ However, the previous study had several limitations that are imperative to understanding the impact of AL on survival. First, multicenter data are often heterogenous in regard to the type of surgery performed, which in this case included transhiatal and three-stage procedures that are established to have different survival profiles. In contrast, the present study focused only on patients who had undergone a two-stage transthoracic esophagectomy. Second, the previous multicenter study is limited by center variation in the context of volume, pathological assessment of specimens, and multidisciplinary pathways.^9^ Interestingly, center volume was associated with SEAL, which may also reflect the poor long-term survival outcomes. In addition, multicenter studies such as this, as well as other smaller studies, make it difficult to adjust for 30- and 90-day mortality. In the present study, all patients went through the same standardized multidisciplinary team process and were treated in a high-volume unit. There has been an increasing trend for esophagectomies to be carried out at high-volume units as this has been proven to improve both short- and long-term outcomes.^20^^–^22

The mechanism by which AL contributes towards poorer long-term survival is unclear. It has been postulated and extrapolated from colorectal surgery studies that cancer cells may be shed into the gut lumen during surgery,^23^ and thus anastomotic leakage allows these to spread into the mediastinum, contributing to local recurrence.^9^ There is conflicting evidence regarding whether perioperative morbidity impacts on long-term survival. While several studies have indicated that complications have no impact on survival,^24^^,^^25^ data from a Swedish national database suggested that surgical complications may be a poor predictor of long-term survival,^26^ and further studies from Japan have implicated pulmonary infections as having an unfavorable prognosis in patients who received neoadjuvant chemotherapy.^10^^,^^27^^,^^28^

In the present study, patients underwent a standardized two-field transthoracic esophagectomy, which has been previously described. Median lymph node yield was high and R1 resection rates low, both of which have been shown to contribute towards improved OS.^29^^,^^30^ There appears to be no difference in the impact of AL, irrespective of whether or not a patient received neoadjuvant treatment. However, it may be that in the era of neoadjuvant treatment, the long-term oncological impact of an AL is lessened by this treatment.

While it could be argued that one of the weaknesses of this study is that all the data come from a single unit, this is also one of its strengths as it provides evidence of potential outcomes for patients when looked after at an experienced center with a high volume of patients. The overall AL rate of 8% is comparable with other studies.^4^^,^^10^^,^^15^^,^^26^^–^29 For those patients who developed a leak, the mortality rate was low (2%) and this translates to a 0.2% chance of dying from an AL after esophagectomy. While it is clear that AL is likely to prolong both critical care stay and the total time spent in hospital, an aggressive conservative management strategy can provide excellent outcomes.^2^

## Conclusion

The present study refutes the suggestion that AL leads to poorer long-term oncological outcomes. A standardized esophagectomy with careful consideration for oncological principles and management of complications at a high-volume center can provide good short- and long-term outcomes after esophagectomy.
